# A systematic analysis of and recommendations for public health events involving brucellosis from 2006 to 2019 in China

**DOI:** 10.1080/07853890.2022.2092894

**Published:** 2022-07-04

**Authors:** Zhiguo Liu, Miao Wang, Yaxin Tian, Zhongqiu Li, Liping Gao, Zhenjun Li

**Affiliations:** a National Institute for Communicable Disease Control and Prevention, Chinese Center for Disease Control and Prevention, Beijing, China; b Ulanqab Center for Disease Control and Prevention, Ulanqab, China; c National Institute of Parasitic Diseases, Chinese Center for Disease Control and Prevention (Chinese Center for Tropical Diseases Research), NHC Key Laboratory of Parasite and Vector Biology, WHO Collaborating Center for Tropical Diseases, National Center for International Research on Tropical Diseases, Shanghai, China; d State Key Laboratory of Infectious Disease Prevention and Control, National Institute for viral Disease Control and Prevention, Chinese Center for Disease Control and Prevention, Beijing, China

**Keywords:** Brucellosis, laboratory *Brucella* infection, brucellosis vaccine infection, China

## Abstract

Brucellosis is a severe public health problem in China. However, analysis on related infection events is lacking. We performed a systematic analysis of brucellosis laboratory infection and vaccine infection events from 2006 to 2019 in China based on the published literatures. Our analysis showed that most laboratory*Brucella*infections in hospitals were found in Southern China. The identification and handling of suspected samples of*Brucella*infection without following the recommended biosafety protection was the main risk factor. It is important to strengthen the preventive awareness of clinical laboratory staff and physicians, while highlighting the compulsory handling and identification of suspected*Brucella*-infected samples in biosafety facilities and following biosafety practices. However, a severe *Brucella* infection accident at the Northeast Agricultural University, with 28 positive cases, showed that strengthening the management in teaching experiments of students in the veterinary-related profession is essential. However, cluster S2 vaccine strain infection events caused by vaccination and production were mainly observed in Northern China. Strengthening vaccination skills, personal protection, and improving the biosafety management of vaccine production and implementing regular risk surveillance is mandatory. Our analysis provides helpful clues for control of public health events involving brucellosis, as well as implementing intervention strategies is urgent.

## Introduction

Brucellosis is one of the most common zoonotic diseases globally [1]. More than 500,000 new human cases of brucellosis are reported annually, and millions of livestock are either infected or at risk [2]. It is a significant public health problem and causes serious harm to the livestock industry’s development and human health [3]. At present, at least 12 *Brucella* species have been identified in the genus *Brucella* [4]. *Brucella melitensis* is a pathogen of goats and sheep and is considered to be the most virulent species for humans [5]. Fever, fatigue, sweating, and muscle and joint pain are the main manifestations in the acute stage of human brucellosis [6]. However, chronic disease can affect various organs, leading to arthritis, orchitis, hepatitis, encephalomyelitis, and endocarditis [7]. *Brucella* spp. are highly infectious because the infectious dose by an aerosol is only 10–100 organisms [8], and it is the most important laboratory-acquired bacterial infection [9]. It is recommended that the organism is handled according to biosafety level 3 (BSL3) precautions [10]. These guidelines can be challenging to follow, particularly in regions with a low incidence of brucellosis.

Laboratory-associated *Brucella* spp. infections may account for up to 2% of all laboratory-associated infections [11]. From 2000 to 2014, 28 cases of brucellosis (median: 2/year) were reported, including 6 *Brucella* exposure risk events (*Brucella* events) in clinical laboratories, resulting in more than 80 occupational exposures to *Brucella* species (spp.) in New York City [12]. Moreover, many studies on laboratory exposure to *Brucella* strains have been reported in the United Kingdom [13] and Denmark [14]. Several brucellosis cases are reported annually in China, but ongoing surveillance of brucellosis infection cases that have occurred in staff from hospitals, universities, and factories is lacking. The epidemiological profile of brucellosis resulting from laboratory transmission of brucellosis in China remains unknown. Moreover, two clusters of brucellosis infection in domestic events have been reported but did not capture the attention of the international scientific community. Hence, in this study, we summarised cases of laboratory-acquired *Brucella* infection in hospitals and universities and cluster vaccine strain infections in China to identify effective countermeasures for preventing laboratory-acquired infections and to promote the design of public health safety measures.

## Methods

### Search strategy

We performed a systematic search across four electronic databases: China National Knowledge Infrastructure (CNKI), Wan Fang Data, Wei Pu Data, and PubMed with the following Mesh terms and keyword subject heading “brucellosis”, “*Brucella*” or their various short terms in Chinese, and “laboratory infection” or “hospital infection”, and “vaccine infection” and “China”; the search fields were Keyword, Abstract, and Full Article; the search languages were Chinese and English; the retrieval time was from the construction of the databases to 2 June 2020. We focussed on studies about the brucellosis laboratory infection and vaccine cluster infection events in China, other aspect studies in brucellosis were all excluded from this analysis. Moreover, we collected the reported vaccine strain outbreak events from the domestic and international scientific community. The flow diagram of literature search and selection is shown in Figure S1.

### Literature screening and data extraction

Reviewers independently extracted and recorded data from each selected study. Information was recorded as follows: the first author, publication Journal (DOI or article code), cases occur time, location of cases, patient demographic profiles, potential transmission route, main symptoms, suspected diagnosis and Final confirmed diagnosis method. We used Excel 2016 (Microsoft, Redmond, WA, USA) for data processing and analysis. The data collection form that was used for this analysis is presented in Table 1.

**Table 1.** Epidemiology and clinical diagnostic characteristics of patients with laboratory-acquired brucellosis cases in hospitals.

	Cases no.	Authors	Journal (DOI or article code)	Time	Location	Patient profiles	Transmission route	Main symptoms	Suspected diagnosis	Final confirmed diagnosis method
La boratory infection events in hospitals (LIE)	LIE_1	Haiyan Zhang, et al.	*Capital Journal of Public Health* (Chinses) (1673—7830(2008)01—0038—02)	2006	Beijing	Male, 45, laboratorian	Identification of suspect *Brucella*	Fever, fatigue, chills, headache	Fever of unknown origin	*Brucella melitensis* obtained and SAT 1:800
	LIE_2	Jianhua Zhou, et al.	*Zhe Jiang Prevernt Medical* (Chinses) (1007-0931(2008)) 09-0026-01	2007	Hangzhou	Female, 34, laboratorian	Handling of suspected samples	Undulant fever, fatigue,	Suspect brucellosis	*Brucella melitensis* obtained and SAT 1:800
	LIE_3	Heng Wang, et al.	*Emerging Microbes & Infection* (DOI:10.1080/22221751.2020.1754137)	2009	Hangzhou	Unknown, laboratorian	Identification of suspect *Brucella*	Unknown	Unknown	*Brucella melitenesis* obtained
	LIE_4			2011	Quzhou	Unknown, laboratorian	Identification of suspect *Brucella*	Unknown	Unknown	
	LIE_5	Tiao Liu, et al.	*Chinese Journal of Epidemiology* (Chinses) DOI:10.3760/cma.jissn.2095-4255.2013.06.031	2011	Suzhou	Female, 49, laboratorian	Identification of suspect *Brucella*	Fatigue, intermittent low fever, headache	Fever of unknown origin	*Brucella melitensis* obtained and SAT 1:400
	LIE_6	Jie Liu, et al.	*Chinese Journal of Industry Medical* (Chinses) (DOI: 10.13631/j. cnki.zggyyx.2014.06.005)	2012		Female, 30, laboratorian	Handling a strain from case 3	Fatigue, low fever, sweating, back pain	Fever of unknown origin	*Brucella* obtained and SAT 1:100
	LIE_7	Shaxia Yu, et al.	*Journal of Ningxia Medical University* (Chinses) (1674-6309(2017)01-0107-03)	2013	Ningxia	Female, 30, laboratorian	Identification of suspect Brucella	Fever of unknown origin, 39.8 °C, sweating	Fever of unknown origin	*Brucella* obtained and SAT 1:100
	LIE_8	Jun Zhang, et al.	*Journal of Medical Pest Control* (Chinses) (10.7629/yxdwfz201409026)	2013	Yangzhou	Female, 49, laboratorian	Handling blood culture samples	Undulant fever, sweating	Fever of unknown origin	*Brucella* obtained and SAT 1:100
	LIE_9	Jingwen Wu, et al.	*Practical Prevent Medical* (Chinses) (DOI:10.3969/j.issn.1006-3110.2018.10.012)	2016	Nanchang	Female, 60, cleaner	Cleaning up the laboratory waste	Fever, fatigue, sweating, lumbago	Inflammation of psoas major muscle	*Brucella* obtained and SAT 1:100
	LIE_10					Male, 65, cleaner	Cleaning up the laboratory waste	High fever of unknown origin, sweating	Fever of unknown origin	*Brucella* obtained and SAT 1:100
	LIE_11	Le Zhang, et al.	*Modern Preventive Medicine* (Chinses) (1003-8507 (2017) 11-076-05)	2016	Hefei	Female, 28, laboratorian	Handling blood culture samples	Undulant fever, sweating, joint pain	Postpartum low fever, viral cold	SAT 1:100 and *Brucella melitenesis* obtained
Accidental infection at a veterinary college (AIV)	AIV_1	Wenjun Li	*Education and Vocation* (Chinses) (DOI:10.3969/j.issn.1004-3985.2011.34.024)	2011	Heilongjiang	27 studetns and 1 lecturer	Animals anatomy experiments class	Fever, joint pain (mostly)	Suspect brucellosis	Anti-*Brucella* antibodies positive
Vaccine infection events (VIE)	VIE_1	Shaojue Liu	*China Animal Health* (Chinses) http://zgdwbj.com/archives/10152	2011	Ulanqab, Inner Mongolia	Male, more than 100 persons	Unknown (field work or S2 vaccine)	Unreported	Suspect brucellosis	Anti-*Brucella* antibodies positive
	VIE_2	Peng Zhang	*Disease Surveillance* (Chinses) DOI:10.3784/j.issn.1003-9961.2018.03.013	2018	Wuwei, Gansu	Male, 51 persons	S2 vaccine	Muscle, large joint pain	Suspect brucellosis	RBPT: positive; SAT: 1:100
	VIE_3	Chinese Academy of Agricultural Sciences	*Livestock Vet Today: Dairy Cows* (https://d.wanfangdata.com.cn/periodical/ChlQZXJpb2RpY2Fs Q0hJTmV3UzIwMjIw NDE1EhdRS1YyMDE5MjAy MDA0MjgwMDQ1ODcx MRoIaGNsZjh2ZjI%3D)	2019	Lanzhou, Gansu	10,528 persons	S2 vaccine	Unsymptoms (largely)	Suspect brucellosis	Anti-*Brucella* antibodies positive

### Selection criteria

Studies in the laboratory (hospital) *Brucella* infection with the following criteria at least were included, both serology and (/or) bacteriology assays were performed according to previously described procedures [12,15]. (A) The Rose-Bengal plate agglutination test (RBPT) was used for primary screening of suspected patients, and the positive serum samples were confirmed further by serum tube agglutination test (SAT) and a serial dilution method; a titre of 1:≥100 was considered positive, whereas the titre of 1:≥50 and more than 1-year course of illness complemented with clinical symptoms also were confirmed as brucellosis cases. (B) The case with a positive culture for *Brucella* spp. was identified as a definitive brucellosis case [16].

## Results

### Laboratory-acquired infections in hospitals

A total of 11 laboratory-acquired infection events were reported during the 2006–2016 period in China (Table 1). Two events were observed in Northern China (Beijing and Ningxia province), and the remaining nine events were found in Southern China (Zhejiang, Jiangsu, Jiangxi, and Anhui provinces). Nine of the patients were microbiological technicians and two were cleaners of a microbiology laboratory. The age range of the nine patients was 28–65 years old, and the mean age was 45 years old; two patients were men, seven were women and the remaining two were unknown. Seven patients were infected during the identification or handling of suspect *Brucella* strains, two by handling blood culture samples from patients with brucellosis, and the remaining individuals were infected while cleaning up the microbiology laboratory waste. All accidental infections occurred because of substandard laboratory safety conditions, manipulations outside biosafety cabinets (BSCs), or the use of inadequate personal protective equipment. All nine cases presented fever; sweating was observed in six cases, fatigue in five cases, and headache in two cases. Only one patient was suspected of brucellosis, seven cases were diagnosed with a fever of unknown origin, and one patient was diagnosed with inflammation of the psoas major muscle, reminding two cases were unknown. The ten patients were first diagnosed with brucellosis by bacteriology test, and a serology test (SAT titre ≥ 1:100) was used only in one case.

### Accidental infection at a veterinary college

From March to May 2011, 28 individuals (27 students and 1 lecturer) were diagnosed with *Brucella* infections in the College of Animal Medicine of Northeast Agricultural University [17]. The field epidemiology survey showed that the infection source was goats without the quarantine inspection that experimental animals require. The goats infected with *Brucella* spp. were used for teaching experiments (obstetric experiment and animal anatomy experiment) five times, but the name of the specific species/biovar of *Brucella* spp. that caused this incident was not published. Four goats (infected with *Brucella* spp.) were found to be the infection source for these cases. Aerosols and contact with infected goats were the main routes of transmission. Failure to comply with the standard experimental norms and ensure that the students follow the biosafety protection-operating procedures were the main reasons for these cluster infections. According to the unified prescription by the expert committee on accident handling, all patients were treated with intravenous doses of combined doxycycline (200 mg once daily), rifampin (500–1000 mg once daily), and levofloxacin (200–400 mg once daily) (or cefoperazone sodium and sulbactam sodium (200–400 mg once daily)) for 6 weeks, followed by oral rifapentine (300 mg once daily) and tetracycline (500 mg once daily) for 40 days. 25 patients were cured, one patient improved, and two patients still showed a small amount of effusion in the joint cavity by MRI.

### S2 vaccine strain infection events

In 2017, a brucellosis outbreak caused by S2 vaccine strain vaccination was reported in Tianzhu County, Gansu Province, China [18]. A total of 206 animal epidemic prevention controllers participated in the immunization work of the S2 vaccine strain vaccination of sheep in Tianzhu county from November to December in 2016. 51 controllers were positive by serological testing, and the rate of infection was 24.8%. All 51 individuals were RBPT-positive, in which 36 cases had SAT scores ≥1:100 and 15 cases had SAT scores ranging from 1:50 to 1:100. The blood samples from all patients were free of *Brucella* strains. The vaccination work did not comply with the biosafety regulations, which was the main reason for these infections, including improper handling in vaccination and limited understanding of the S2 vaccine strain’s pathogenicity, inadequate personal protection, and imperfect emergency measures. Fatigue and sweat were present in 94.13% of the cases (48/51), fever in four cases, and swelling of the testis in five cases. All patients (all men) were treated with various combinations of antibiotics. The antibiotics regimens (combined doxycycline and rifampin) were given based on China’s guidelines for the diagnosis and treatment of human brucellosis [19]. The therapy was sustained for 4 weeks to 6 months based on the illness situation of the patients. All patients greatly improved.

### Cluster brucellosis vaccine infection events in factories

On 28 November 2019, two students from the prevention and control technology team of Lanzhou Veterinary Research Institute of the Chinese Academy of Agricultural Sciences were serologically positive for *Brucella* spp. Until 25 December 2019, 671 serum samples from the Lanzhou Animal Research Institute students and staff were screened. It was found that 181 persons were positive for anti-*Brucella* antibodies, and only one person had clinical symptoms. Furthermore, 13 people from the Heilongjiang province in Northeastern China who worked at the veterinary institute in August 2019 tested positive for anti-*Brucella* antibodies. Based on the investigation and many field test results, it was concluded that the Zhongmu Lanzhou biopharmaceutical plant had used expired disinfectants from July to August 2019 to make brucellosis vaccines (S2 vaccine strain and A19 vaccine strain), leaving the bacteria in their waste gas, but definite vaccine resulting in this event had not been published. The contaminated gas later formed aerosols that drifted downwind to the veterinary institute. Up to 30 November 2020, 10,528 residents living in the vicinity of Lanzhou Veterinary Institute were positive for anti-*Brucella* antibodies [20]. A total of 3244 people had signed compensation agreements [21]. This left some Lanzhou residents facing chronic illness [22], but the details of the patients’ situations were not published. The government sector has instructed the Zhongmu Lanzhou biopharmaceutical plant to focus on the brucellosis vaccine production facility to immediately conduct a comprehensive inspection on the implementation of quality management specified for veterinary medicine in the whole plant and to make proper rectifications within a time limit. Without approval from the industry’s competency department, the brucellosis vaccine workplace should not resume production.

## Discussion

Brucellosis is the most common bacterial laboratory-acquired infection worldwide [23]. Clinical laboratory workers have failed to recognize suspicious isolates and have manipulated unknown isolates on open benches, using procedures that aerosolized *Brucella*, increasing their exposure risk to biological hazards [24]. In this study, nine laboratory-acquired infection events were found in the southern region, a brucellosis-emerging area [25]. In the southern regions, clinical microbiology laboratories are frequently unfamiliar with the *Brucella* genus, and there is a low index of suspicion by physicians or failure to notify the laboratory that the handled specimens might yield a hazardous organism. Typically, clinicians do not consider brucellosis until notified that bacteraemia with *Brucella* was suspected.

Moreover, the misidentification of the organism by commercial systems, unsafe laboratory practices, and laboratory accidents have been responsible recently for many cases of exposure to the organism and laboratory-acquired disease [26]. In the United States, fewer than 150 cases of brucellosis have been reported annually since 1986, but brucellosis is among the most commonly reported laboratory-acquired bacterial infections [27]. In this study, the problematic current practices when handling blood culture bottles from patients with fever of unknown origin were the main reason for these infection events, and the transmission in our cases was probably due to aerosol contamination. Similarly, most critical exposures involved catalase testing or isolate vortexing, both of which may generate infectious aerosols [28]. In New York City, more than 200 occupational exposures occurred because of the generation of infectious *Brucella* spp. aerosols [24]. In our study, we observed a high risk of developing laboratory-acquired brucellosis in microbiological laboratory workers. In Spain, a total of 75 workers have had laboratory-acquired brucellosis, 57% (43/75) of whom were microbiologists [29]. Thus, we are suggesting that clinicians’ ability to diagnose brucellosis should be improved, especially in the southern region of China. Furthermore, it is essential to promote BSL3 facilities when handling specimens from patients suspected of *Brucella* spp. infection and unidentified isolates. Additionally, prompt assessment of the risk of exposure, information sharing, and postexposure prophylaxis protocols as described previously [30] are recommended. Finally, we consider that the use of personal protective equipment (gloves, masks, and goggles), a BSC, and the continuous strengthening of education regarding biosafety of individuals with high infection risk are significant for preventing laboratory-acquired brucellosis events.

S2 vaccine strain has been widely used in China since 1971 to vaccinate sheep and goats, as it does not lead to the abortion of pregnant females [31]. In this study, many individuals infected with the S2 vaccine strain had manifestations, and few of them had no symptoms. These data suggest that the S2 vaccine strain is virulent for humans because of the strong immune response seen in infected individuals. One study confirmed that the S2 vaccine strain was able to infect and proliferate to high titres, hamper the proliferation of goat trophoblast cells, and induce apoptosis because of endoplasmic reticulum stress [32]. We considered that further investigation into the pathogenicity of the S2 vaccine strain to humans is necessary. Moreover, the Brucella *abortus* S19 vaccine strain was isolated from four persons; all four infected individuals were employed as milkers and did not have clear manifestations of the disease [33]. Although some brucellosis vaccine strains may be transmitted to the human population and may persist in the blood sometimes without causing overt disease [33], protective measures remain important to prevent exposure to vaccine strains. Moreover, more than 100 grassroots animal epidemic prevention officers from Ulanqab of Inner Mongolia were diagnosed with brucellosis seropositivity for a short-time period [34]. They had collected blood samples from ruminants for brucellosis tests and vaccinated and fed the sheep themselves. Moreover, it is well known that Ulanqab is a severe human brucellosis epidemic region [35], and because the conventional serological tests used did not permit discrimination of infected animals from vaccinated animals, further investigation of this event is necessary. We have suggested that the animal husbandry and veterinary department should strengthen the protection knowledge of brucellosis and conduct vaccination according to biological safety regulations. Moreover, the use of expired disinfectants in the production process of the brucellosis vaccine caused numerous individuals in a Zhongmu Lanzhou biopharmaceutical plant to be seropositive for brucellosis. Another study showed that active brucellosis was diagnosed in 21 of 30 employees from vaccine-manufacturing plants, of whom only five recalled an accidental exposure [36]. We suggest improving biosafety management of vaccine production and implementing regular risk surveillance and a training program to reduce the occupational infection of *Brucella* spp. strain are urgent measures.

The isolation of *Brucella* is time-consuming, requires skilled technicians, and represents a high risk to laboratory personnel [37]. To date, no tests that can help differentiate between vaccinated and naturally infected individuals have been officially approved. At present, a series of molecular methods was employed for the early and rapid diagnosis of infectious diseases, such as quantitative polymerase chain reaction [38]. Moreover, next-generation sequencing holds the potential for improving clinical and public health microbiology [39], and laboratories may be able to replace many traditional microbiology processes with a single workflow that accommodates a wide array of pathogens [40], which will prevent occupational *Brucella* spp. infection. We recommend evaluating the feasibility and reproducibility of this approach and its approval as rapid tools for the official diagnosis of brucellosis.

Our study has some limitations. The settings in which each of these types of exposure occurred were varied, and we lack detailed epidemiological data for various cases. The conclusions reported here are partially trusted to reflect the infection situation of these cases. Moreover, detailed information was only found for the S2 vaccine exposure case, and information regarding the type of “accident” or exposure that caused these infections (needle stings, conjunctival splashes, and broken flasks) is lacking, further related survey is warranted. Finally, our present data is unsupported for implementing a meta-analysis of public health events involving brucellosis, so a detailed field investigation of brucellosis laboratory infection on a country scale is recommended for better form the interview strategies.

## Conclusion

This report serves as a reminder that occupational *Brucella* exposure is a risk among clinical laboratory staff and veterinary personnel. Moreover, the lack of prevention knowledge in high-risk populations and failure to follow good biosafety practices are important risks for transmission of occupational *Brucella* spp. infection. This comprehensive summary of the *Brucella* spp. strain infection events in hospitals, universities, and factories improve our understanding of the epidemiology of brucellosis in China. Furthermore, improper use of protective measures also increases the probability of infection. Given that human brucellosis cases continue to spread widely [25], the prevention of *Brucella* spp. infection in hospitals, universities, and vaccine factories remains a considerable challenge.

## Supplementary Material

Supplemental Material

## Data Availability

All data generated or analyzed during this study are included in our article.

## Abbreviations

BSL3: biosafety level 3 (BSL3)
BSC: biosafety cabinet
RBPT: Rose-Bengal Plate Agglutination Test
SAT: Serum Tube Agglutination Test
