# Supplementary figures and images for: A systematic analysis of and recommendations for public health events involving brucellosis from 2006 to 2019 in China
Source: Ann Med. 2022 Jul 4;54(1):1859–66. doi: 10.1080/07853890.2022.2092894 (PMC9258428; doi:10.1080/07853890.2022.2092894)

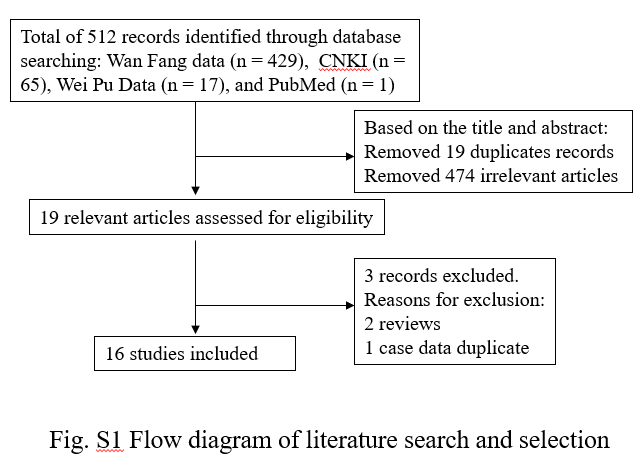

Supplement: Supplemental Material [file IANN_A_2092894_SM2948.tif]
